# Identification of m6A Modification Regulated by Dysregulated circRNAs in Decidua of Recurrent Pregnancy Loss

**DOI:** 10.3390/cimb45110551

**Published:** 2023-10-31

**Authors:** Liyuan Cui, Minfeng Shi, Xinhang Meng, Jinfeng Qian, Songcun Wang

**Affiliations:** 1 Laboratory for Reproductive Immunology, Hospital of Obstetrics and Gynecology, Shanghai Medical College, Fudan University, Shanghai 200090, China; lycui15@fudan.edu.cn (L.C.); 22211250012@fudan.edu.cn (X.M.); 2 State Key Laboratory of Medical Neurobiology, MOE Frontiers Center for Brain Science, Fudan University, Shanghai 200032, China; 3 Reproductive Medicine Center, Changhai Hospital, Naval Medical University, Shanghai 200433, China; minfengshi@163.com

**Keywords:** recurrent pregnancy loss, decidua, circRNAs, ceRNA network, m6A modification, serum

## Abstract

N6-methyladenosine (m6A) modification is a prevalent modification of messenger ribonucleic acid (mRNA) in eukaryote cells and is closely associated with recurrent pregnancy loss (RPL). Circular RNAs (circRNAs) play critical roles in embryo implantation, trophoblast invasion and immune balance, which are important events during pregnancy. However, how m6A modification is regulated by circRNAs and the potential regulatory mechanism of circRNAs on RPL occurrence remain largely unclassified. We displayed the expression profiles of circRNAs and mRNAs in the decidua of normal pregnancies and RPL patients based on circRNA sequencing and the Gene Expression Omnibus database. A total of 936 differentially expressed circRNAs were identified, including 509 upregulated and 427 downregulated circRNAs. Differentially expressed circRNAs were enriched in immune, metabolism, signaling and other related pathways via the analysis of Gene Ontology (GO) and the Kyoto Encyclopedia of Genes and Genomes (KEGG) pathway. The competitive endogenous RNA (ceRNA) network was predicted to supply the possible role of circRNAs in RPL occurrence, and we further analyzed the profiles of nine m6A regulators (seven readers, one writer and one eraser) managed by circRNAs in this network. We also showed the expression profiles of circRNAs in the serum, trying to seek a potential biomarker to help in the diagnosis of RPL. These data imply that circRNAs are involved in pathogenesis of RPL by changing immune activities, metabolism and m6A modification in the ceRNA network. Our study might provide assistance in exploring the pathogenesis and diagnosis of RPL.

## 1. Introduction

Recurrent pregnancy loss (RPL) refers to the failure of two or more consecutive clinical recognized pregnancies prior to 20–24 weeks of gestation, which is frustrating for about 1–5% of couples [1]. The etiology of up to 50% of RPL patients remains unexplained, although chromosomal abnormalities, immune dysfunction, endocrine diseases, endometrial dysfunction, and environmental and psychological factors have been established as causes of RPL. The decidua is critical for the establishment and maintenance of pregnancy, and it secretes various factors to participate in the regulation of implantation, immune tolerance and trophoblast differentiation [2]. Misfunctions of decidua, such as immune imbalance and metabolic disorders, are significant causes of RPL [3,4,5]. Recent research revealed the relationship between epigenetic changes, such as dysregulated noncoding RNAs in decidua, and RPL [6]. RPL is an extremely complex health issue globally; thus, exploring more detailed epigenetic factors is highly necessary.

Circular ribonucleic acids (circRNAs), as a class of endogenous noncoding RNAs, are generated through backsplicing, characterized by a covalently linked loop structure without 5′ caps and 3′ tails, and are more stable than other linear RNAs [7,8]. CircRNAs play important roles in various biological processes by functioning as micro RNA (miRNA) sponges, transcriptional regulators, regulators of alternative splicing and others [9,10]. Previous research has proven the existence of circRNAs in decidua, placentae, villus and ovaries, which are involved in the regulation of implantation and function of trophoblast cells by sponging miRNAs [9]. A competitive endogenous RNA (ceRNA) network prediction, based on the differential expression of circRNAs, miRNAs and messenger RNAs (mRNAs), are used to evaluate the roles of circRNAs in several biological processes. Previous studies revealed that the proliferation, migration and invasion of trophoblast cells were regulated by Circ_0074371-miR-582-3p-LPR6, Circ_0011460-miR-762-HTRA1, Circ_0037078-miR-576-5p-IL1RAP and Circ_0017068-miR-330-5p-XIAP axes [11,12,13,14]. Macrophage proinflammatory polarization was regulated by the circKIAA0391-miR-512-5p-MITA axis [15]. Therefore, disturbed circRNA expression in villus and decidua may be one cause of RPL. However, the specific roles of decidual circRNAs in RPL are still not entirely clear.

N6-methyladenosine (m6A) modification, known as an abundant RNA modification, is modulated by corresponding enzymes, including methyltransferases, such as Wilms tumor-1-associated protein (WTAP), methyltransferase-like 3 (METTL3) and methyltransferase-like 14 (METTL14); demethylases, such as the fat mass and obesity-associated protein (FTO) and alkB homolog 5 (ALKBH5); and m6A-binding proteins, such as the YT521-B homology domain family (YTHDFs), referred to as “erasers”, “writers” and “readers”, respectively [16,17]. m6A modification affects mRNA stability, splicing, translation and transport and is involved in various biological processes, including immune responses and metabolic functions. Recent research has proven that m6A modification aberrancies could damage trophoblast invasion, decidual stromal cell function and immunotolerance, whose dysfunction could cause RPL [18,19,20]. However, whether m6A modification is regulated by circRNAs during RPL has not been fully explained.

In this study, expression profiles of circRNAs in decidua from RPL patients and normal pregnancies were explored first. We then tried to clarify the role of circRNAs in RPL occurrence. The regulatory mechanism of circRNAs in RPL occurrence was predicted via the ceRNA network and m6A modification analyses. Moreover, the expression profiles of circRNAs in serum of peripheral blood were also analyzed to help find a clinical biomarker for RPL.

## 2. Materials and Methods

### 2.1. Human Sample

Human decidual tissues and peripheral blood from first-trimester human pregnancies were obtained from RPL patients (RPL group) and clinically normal pregnancies (NP group: terminated for nonmedical reasons (elective terminations of healthy pregnancies), had at least one healthy child and had no history of spontaneous abortions). The clinical characteristics of the enrolled subjects are summarized in Table 1. The subjects with RPL included those undergoing spontaneous abortion and also had a history of two or more consecutive spontaneous abortions without known causes (including parental or embryonic karyotype anomalies, uterine anatomic abnormalities, infection-associated factors, endocrine disorders, antiphospholipid syndrome, etc.). None of the subjects had any history of autoimmune disease, immunotherapy, hormone therapy, renal or liver diseases, alcohol addiction, smoking or vaccination within 3 months before sample collection.

In total, six paired decidua and peripheral blood serum samples from the NP and RPL groups were collected for circRNA sequencing (circRNA-seq). Six deciduae from the NP group and six deciduae from the RPL group were collected for real-time quantitative reverse-transcription polymerase chain reaction (RT-PCR) validation. All participants gave written informed consent. All performances were in compliance with the Human Research Ethics Committee of the Obstetrics and Gynecology Hospital of Fudan University (approval No. Kyy2021-11).

### 2.2. CircRNA-Seq

The total RNA was extracted from the sera and decidual tissue using Takara RNAiso Plus, according to protocol. The concentration and integrity of the extracted total RNA were estimated with a Qubit 3.0 Fluorometer (Invitrogen, Carlsbad, CA, USA) and an Agilent 2100 Bioanalyzer (Applied Biosystems, Carlsbad, CA, USA), respectively. For tissue sequencing, a library was prepared using a KAPA RNA HyperPrep Kit with RiboErase (HMR) for Illumina^®^ (Kapa Biosystems, Inc., Woburn, MA, USA). Briefly, the ribosome-depleted RNA was incubated for 30 min at 37 °C with 10 units of RNase R (Epicentre Technologies, Madison, WI, USA) and purified with VAHTS RNA Clean Beads. Then, the ribominus RNase R (+) RNAs were fragmented and first-strand and directional second-strand syntheses were performed. Next, A-tailing and adapter ligation were performed with the purified cDNA. Finally, the purified and adapter-ligated DNAs were amplified. For sequencing, a library was prepared using a Clontech SMARTer Stranded Total RNA seq kit with V2 pico input (Takara, Tokyo, Japan). Briefly, the RNAs were fragmented; then, first-strand synthesis was performed with the Template Switching Oligo Mix. Subsequently, the addition of adapters and indexes was carried out with first-round PCR. Next, ribosomal cDNA was depleted with ZapR and R-Probes. Finally, library amplification was performed with the recommended cycles. The quality and concentration of the library were assessed utilizing a DNA 1000 chip on an Agilent 2100 Bioanalyzer. The qualified libraries were sequenced using a Novaseq 6000 in PE150 mode.

### 2.3. CircRNA–miRNA–mRNA Network Construction

We used miRanda, TargetScan and RNAhybrid to predict the miRNA binding sites of differentially expressed circRNAs, with set values of free energy of <−15 and an alignment score of ≥140. The miRNA data were obtained from miRBase v21. If a circRNA–miRNA relationship was predicted using the three software products simultaneously, it was retained for subsequent analysis. Subsequently, we used multi-MiR Bioconductor software to perform miRNA target prediction based on the miRNAs predicted using the circRNA–miRNA relationships and the differentially expressed mRNAs in the Gene Expression Omnibus (GEO) database. We only retained the potentially conservative miRNA gene relationship, which needed to be predicted with at least five prediction softwares. For prediction of the miRandaTargetScan/pita conservative relationship, at least one record was recorded in any software. Then, we used the Bioconductor software dusterProfiler to perform Gene Ontology (GO)/Kyoto Encyclopedia of Genes and Genomes (KEGG)/Reactome functional and pathway enrichment analysis on the target genes of the miRNAs.

### 2.4. RT-PCR

The circRNAs from the deciduae were extracted using a Fast Tissue RNA Purification Kit (EZB-RN5; EZBioscience, Suzhou, China), following the manufacturer’s instructions. The RNA concentration was detected with a microspectrophotometer (Nanodrop One 190; Thermo Fisher Scientific, Carlsbad, CA, USA), and 1000 ng of RNA was reverse-transcribed into cDNA using a PrimeScript™ RT reagent Kit (RR037A; Takara, Tokyo, Japan) for 15 min at 37 °C, followed by 5 s at 85 °C on a PCR thermocycler (Mastercycler Pro, Eppendorf, Hamburg, Germany). The cDNA was amplified with SYBR Green PCR Master Mix (EZBioscience, Suzhou, China) on an ABI PRISM 7900 Sequence Detection System (Applied Biosystems, Waltham, MA, USA). The Hot Start DNA Polymerase required brief activation at 95 °C for 5 min. Then, 40 cyclic reactions were performed. The reaction system was denatured at 95 °C for 10 s and then annealed/extended at 60 °C, with 30 s for each cyclic reaction. Fluorescence signals were collected during the annealing/extension step. ACTB was used as an internal control to normalize the relative changes in gene expression with the 2^−∆∆Ct^ method. The primers for the genes are listed in Table 2. The primers for the ACTB were purchased from Sangon Biotech.

### 2.5. Statistical Analysis

Data are shown as means ± standard errors of the mean (SEMs). Statistical differences were analyzed using GraphPad Prism version 7. Normal distribution was tested using the Shapiro–Wilk test. Student’s two-tailed t-test and a Mann–Whitney test were applied to analyze parametric and nonparametric data, respectively. *p* < 0.05 was considered a statistically significant difference.

## 3. Results

### 3.1. Expression Profiles of circRNAs and mRNAs in Decidua from RPL Patients

**Figure 1:**
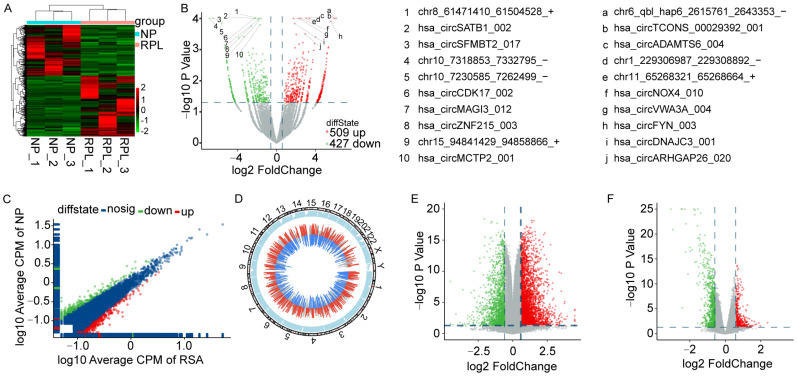
Expression profiles of circRNAs and mRNAs in deciduae from patients with RPL. (**A**) Clustering heat map of differentially expressed circRNAs in the NP and RPL groups. (**B**,**C**) Volcano plot (**B**) and scatter plot (**C**) of circRNAs in the NP and RPL groups. The top 10 upregulated and downregulated circRNAs are labeled in B. (**D**) Chromosome distribution of differentially expressed circRNAs. (**E**,**F**) Volcano plot of mRNAs in GSE26787 (**E**) and GSE165004 (**F**). Red dots represent upregulated mRNAs and green dots represent downregulated mRNAs. NP represents normal pregnancy and RPL represents recurrent pregnancy loss.

Hierarchical clustering was performed to reveal the differentially expressed circRNAs in deciduae from normal pregnancies (the NP group) and patients with RPL (the RPL group) after stringent filtering with the screening criteria of fold change (FC) of ≥1.5 and a *p*-Value of <0.05. A total of 936 differentially expressed circRNAs were identified, 509 of which were upregulated and 427 downregulated (Figure 1A). Volcano and scatter plots were applied to show the variability between the NP and RPL groups (Figure 1B,C). The top 10 upregulated and downregulated circRNAs are shown in Figure 1B. The differentially expressed circRNAs were widely distributed on all chromosomes (Figure 1D). Most of these were exon–exon circRNAs whose lengths were shorter than 1000 bp (Figure S1A,B). The differentially expressed mRNAs were assessed based on GSE26787 [21] and GSE165004 (https://www.ncbi.nlm.nih.gov/geo/query/acc.cgi?acc=GSE165004, accessed on 18 January 2021), and according to the screening criteria of FC ≥ 1.2 and *p* < 0.05. In total, 7823 differentially expressed mRNAs (5830 upregulated and 1993 downregulated) were identified based on GSE26787 (Figure 1E), and 6934 such mRNAs (3355 upregulated and 3579 downregulated) were identified based on GSE165004 (Figure 1F). In total, 12011 differentially expressed mRNAs (7514 upregulated and 4497 downregulated) were obtained according to the union of differential genes between the two databases. These data revealed that the circRNA and mRNA expressions in the deciduae of the RPL group were different from those in the NP group. 

### 3.2. GO and KEGG Pathway Analyses of Parental Genes of Differentially Expressed circRNAs in Decidua

**Figure 2:**
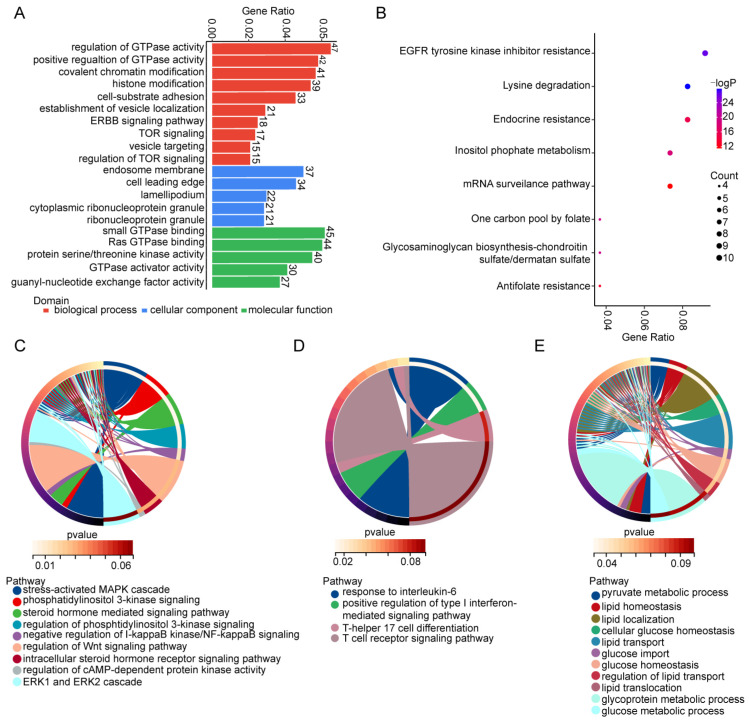
GO and KEGG analyses of parental genes of differentially expressed circRNAs. (**A**) The enrichment analysis of parental genes of differentially expressed circRNAs in the three categories of GO term. (**B**) KEGG analysis of parental genes of differentially expressed circRNAs. (**C**–**E**) Chord plot of signaling (**C**), immune-related (**D**) and metabolism-related (**E**) pathways in biological process of GO enrichment analysis of parental genes of differentially expressed circRNAs.

To obtain further insights into the biological function of dysregulated circRNAs in the RPL group, GO and KEGG analyses were performed. GO term enrichment includes three categories: biological processes, cellular components and molecular functions. The top 10 pathways in the biological process category were those for the regulation of guanosine triphosphatase (GTPase) activity, the positive regulation of GTPase activity, covalent chromatin modification, histone modification, cell–substrate adhesion, establishment of vesicle localization, erythroblastic oncogene B (ERBB) signaling, target of rapamycin (TOR) signaling, vesicle targeting and the regulation of TOR signaling (Figure 2A), the dysfunction of which may damage the roles of the membranes, cytoplasm and nuclei of decidual cells. The enriched KEGG pathways were those for epidermal growth factor receptor (EGFR) tyrosine kinase inhibitor resistance, lysine degradation, endocrine resistance, inositol phosphate metabolism, mRNA surveillance, one carbon pool by folate, glycosaminoglycan biosynthesis–chondroitin sulfate/dermatan sulfate and antifolate resistance (Figure 2B). The related parental genes are listed in Table S1. Cellular signaling pathways, immune responses and metabolic functions play significant roles in the establishment and maintenance of pregnancy; the related pathways in that biological process category were selected for further study (Figure 2C–E). Recent research has reported that the mitogen-activated protein kinase (MAPK), phosphatidylinositol 3-kinase (PI3K), wingless/integrated (Wnt) and extracellular regulated protein kinase (ERK) signaling pathways participate in the regulation of trophoblast invasion and migration [22,23]. PI3K/NF-kappaB/cAMP-related signaling pathways are involved in the regulation of immune tolerance [24]. Decidualization could also be regulated by the PI3K/Wnt/nuclear factor kappa-B (NF-kappaB), cyclic adenosine monophosphate (cAMP) and steroid hormone signaling pathways [25,26,27]. Therefore, several dysregulated circRNAs might play important roles in trophoblast invasion and migration, immune tolerance and decidualization. Some parental genes of dysregulated circRNAs are enriched in the interleukin-6/type I interferon, T-helper 17 cell differentiation and T-cell receptor-related signaling pathways (Figure 2D), which are significant in the maintenance of maternal–fetal tolerance. Metabolic disorders are also related to RPL [28,29], but the underlying mechanisms thereof have not been clarified clearly. The parental genes of dysregulated circRNAs are enriched in metabolic pathways, such as those that are pyruvate-, lipid- and glucose-related. These results suggest that circRNAs might participate in the pathogenesis of RPL by damaging decidual cell functions via cellular signaling and immune/metabolism-related pathways.

### 3.3. Prediction of ceRNA Network and Functional Analyses of m6A Modification in This Network

**Figure 3:**
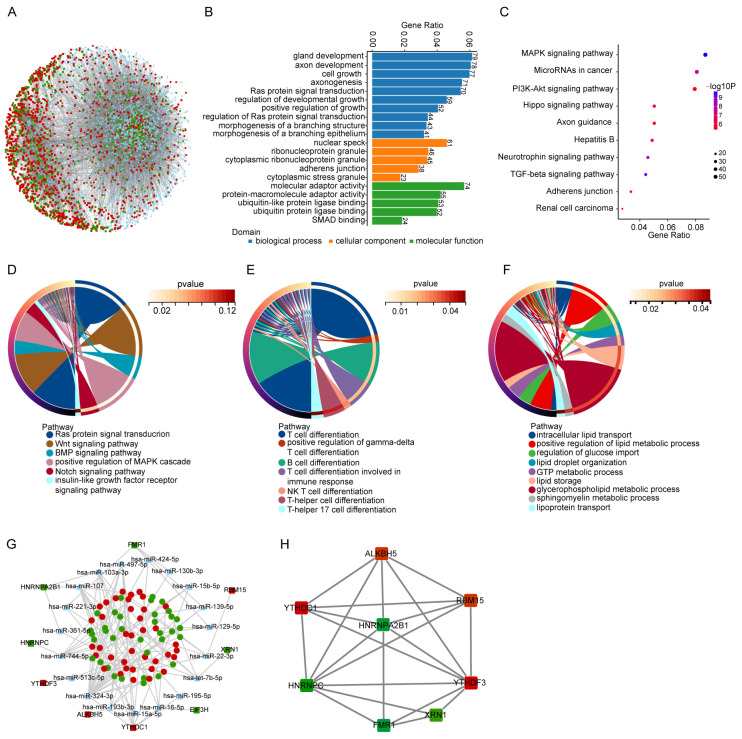
Prediction of ceRNA network and m6A modification analysis of differentially expressed circRNAs. (**A**) ceRNA network map based on differentially expressed circRNAs in microarray analysis and mRNA in GEO databases. (**B**) Enrichment analysis of mRNAs in the ceRNA network in the three GO term categories. (**C**) KEGG analysis of mRNAs in the ceRNA network. (**D**,**F**) Chord plots of signaling (**D**), immune-related (**E**) and metabolism-related (**F**) pathways in the biological process of GO enrichment analysis of mRNAs. (**G**) ceRNA network map based on differentially expressed circRNAs in circRNA-Seq and m6A regulators in GEO databases. Red dots represent upregulated mRNAs and green dots represent downregulated mRNAs. (**H**) PPI network of m6A regulators in the ceRNA network. Red squares represent upregulated m6A regulators and green squares represent downregulated m6A regulators.

The expressions of circRNAs and mRNAs in the deciduae were different between the NP and RPL groups. CircRNAs, as miRNA sponges, regulate miRNA transcription and then change the expression of downstream mRNAs [9]. To further clarify the connection between dysregulated circRNAs and mRNAs in the decidua, a ceRNA network was obtained using combined circRNA–miRNA and miRNA target prediction. The ceRNA network was composed of 882 circRNAs, 1738 miRNAs and 1364 mRNAs (Figure 3A). GO and KEGG analyses were performed to estimate the function of the mRNAs in this network. GO analysis of the biological processes suggested that the dysregulated mRNAs were mainly enriched in the developmental and signal-transduction-related pathways (Figure 3B). Most of the top 10 pathways in the KEGG enrichment analysis were signaling-related (Figure 3C). Similarly, the GO terms in the biological process category were analyzed regarding the aspects of signaling, immune-related and metabolism-related pathways. The enriched pathways of dysregulated mRNAs in these three aspects were almost similar to those of dysregulated circRNAs (Figure 3D–F).

To refine the functions of these mRNAs and construct the ceRNA network, we selected those that were characterized as m6A regulators. This network was composed of 73 circRNAs, 19 miRNAs and nine mRNAs, the last of which included one writer (RBM15), one eraser (ALKBH15) and seven readers (FMR1, XRN1, YTHDF3, HNRNPA2B1, HNRNPC, YDHC1 and EIF3H) (Figure 3G). Protein–protein interaction (PPI) networks were constructed to show the interactions of the m6A regulators (Figure 3H). Interactions were observed among writers, erasers and readers. YTHDF3 and HNRNPC were the top two most highly interrelated mRNAs with others (Figure 3H). These results suggest that m6A modifications are regulated by circRNAs and play significant roles in pregnancy.

### 3.4. Validation of Differentially Expressed circRNAs

**Figure 4:**
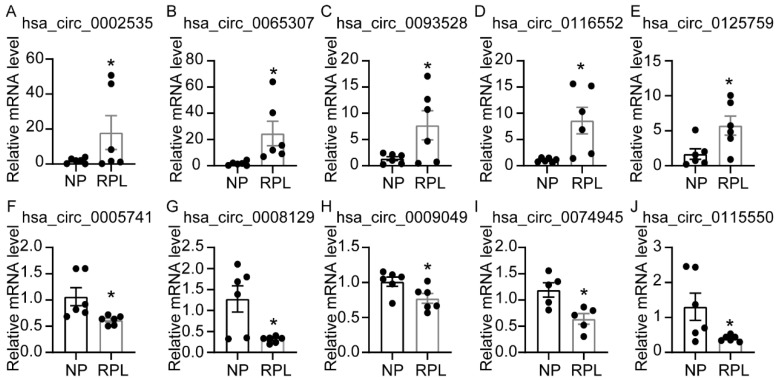
Relative mRNA expression of circRNAs in deciduae of NP and RPL groups. (**A**–**E**) Relative mRNA expressions of hsa_circ_0002535, hsa_circ_0065307, hsa_circ_0093528, hsa_circ_0116552 and hsa_circ_0125759, which were upregulated in the RPL group compared to the NP group. (**F**–**J**) Relative mRNA expressions of hsa_circ_0005741, hsa_circ_0008129, hsa_circ_0009049, hsa_circ_0074945 and hsa_circ_0115550, which were downregulated in the RPL group compared with the NP group. Circles represent raw data. Data are shown as means ± SEMs. * *p* < 0.05.

The expressions of the 10 circRNAs related to m6A regulators were confirmed using RT-PCR. Those of hsa_circ_0002535, hsa_circ_0065307, hsa_circ_0093528, hsa_circ_0116552 and hsa_circ_0125759 were upregulated (Figure 4A–E), and those of hsa_circ_0005741, hsa_circ_0008129, hsa_circ_0009049, hsa_circ_0074945 and hsa_circ_0115550 downregulated in the deciduae of the RPL group compared with those in the NP group (Figure 4F–J). These expression trends matched the results of the circRNA-seq.

### 3.5. Prediction of Biomarker in Serum of Peripheral Blood

**Figure 5:**
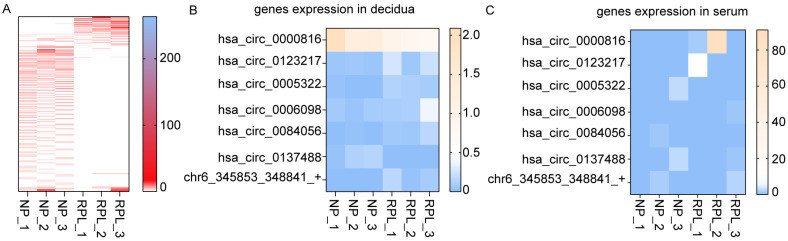
Expression profiles of circRNAs in serum. (**A**) Heat map of circRNAs in the NP and RPL groups. (**B**) Heat map of seven circRNAs in the deciduae of the NP and RPL groups. (**C**) Heat map of seven circRNAs in sera of the NP and RPL groups.

To identify whether the differentially expressed circRNAs could be regarded as blood biomarkers for RPL, the expression profiles of the circRNAs in the serum of peripheral blood were analyzed. The expressions thereof varied between the NP and RPL groups but displayed no statistically significant difference (Figure 5A). Seven differentially expressed decidual circRNAs (Figure 5B) were also detected in the serum (Figure 5C). The expressions of hsa_circ_0123217, hsa_circ_0006098 and chr6_345853_348841_+ had increasing trends and the expression of hsa_circ_0137488 had a decreasing trend in the sera from the RPL group compared with that from the NP group. These expression trends are similar to those in the deciduae. The expression of hsa_circ_0000816 in the RPL serum was upregulated, but its expression in the deciduae of the RPL group was decreased compared with that in the NP group. The expressions of hsa_circ_0005322 and hsa_circ_0084056 in the serum of the RPL group were downregulated, but their expressions in the deciduae of the same group were increased compared with those in the NP group (Figure 5B,C). The sample size should be expanded to further detect expression changes in seeking a reliable biomarker for RPL.

## 4. Discussion

CircRNAs, characterized as having a closed-loop structure, are resistant to exonuclease RNase R. Recent research has suggested that circRNAs play important roles in the pregnancy process and that their abnormal expression or dysfunction could result in the occurrence of pregnancy-related diseases such as pre-eclampsia, gestational diabetes mellitus and RPL [7,30]. Most studies have displayed the expression profiles of the circRNAs in patients with pre-eclampsia, and several studies have shown the expression profiles of the circRNAs in patients with miscarriages [6,10,31]. However, the functional analysis and pathogenesis of decidual circRNAs during RPL remain largely unclarified. In this study, we displayed the expression profiles of circRNAs in the deciduae of RPL patients and analyzed the main functions of the parental genes of the circRNAs regarding the aspects of signaling, metabolic-related and immune-related pathways using GO and KEGG analyses. Combined with the mRNA information in the GEO database, ceRNA network prediction and m6A modification were performed to predict the potential function of dysregulated circRNAs in RPL. The expression profiles of the circRNAs in the sera of patients with RPL were also compared with those in the deciduae to provide supports that would help to find biomarkers for the diagnosis and treatment of RPL. 

The different functions of circRNAs, as miRNA sponges and transcriptional regulators, determined the diversity of their mechanisms involved in the pregnancy process. CircRNAs could also regulate the expressions of parental genes [9]. Little attention was paid to the effect of circRNAs on immune responses. Retinoic acid receptor-related orphan receptor alpha (RORA) is a negative immune reaction regulator. It could regulate the macrophage polarization, T-helper (Th)17 development and inflammatory response of CD4^+^ T cells [32,33]. As a parental gene of hsa_circ_0104051, hsa_circ_0104053 and hsa_circ_0104054 in the present study, it was enriched in the Th17 cell differentiation pathway in the GO term biological process analysis. When we combined this with the analysis of GSE26787 and GSE165004, we found that the mRNA levels of RORA in deciduae from RPL patients were decreased compared with those from normal pregnancies. Additionally, RORA was predicted, using ceRNA network analysis, to be controlled by the hsa_circST7L_026–hsa-miR-1288-3p, hsa_circNDUFS1_010/hsa_circMETTL6_006/hsa_circFAM13B_024–hsa-miR-18b-5p and hsa_circATG7_033/hsa_circMETTL6_006–hsa-miR-18a-5p axes. These data suggest that RORA expression might be regulated directly by circRNAs or indirectly by the ceRNA network and then participate in immune regulation during pregnancy. Therefore, a dysregulated gene in RPL patients might be coregulated by various circRNAs. However, the specific regulatory mechanism of RORA on immunity during pregnancy needs further exploration.

As the most abundant modification of internal mRNA in mammalian cells, m6A modification plays important roles in the splicing, translation, stability and export of mRNAs, which determines their participation in many biological processes [16,34]. Emerging research has suggested that m6A modification plays critical roles in embryo implantation, trophoblast invasion and immune tolerance, which are important events in the pregnancy process [19,35]. Three kinds of m6A regulator (writers, erasers and readers) contribute to m6A modification. We made a further insight into the corresponding roles and found that nine m6A regulators can be managed by circRNAs. YTHDF3, as an m6A reader, can initiate circRNA translation [36], and our data show that it can be regulated by the hsa_circCNTRL_023/hsa_circNPHP4_005–hsa-miR-513c-5p axis. Moreover, it interacted with seven other m6A regulators but not EIF3H. Both YTHDC1 and EIF3H were regulated by the hsa_circAKR1A1_007–hsa-miR-16-5p axis. Therefore, m6A regulators might be regulated by multiple circRNAs and other m6A regulators, and which one has the greatest effect on RPL occurrence is still unclear. Further study exploring the downstream genes that are regulated by m6A regulators during RPL is needed.

The clinical prediction of RPL is limited to low-specificity biomarkers; a noncoding RNA-based diagnosis might be a new choice. Because of their covalently closed loop structure, circRNAs are not as easily degraded by RNase R compared with other noncoding RNAs [16]. Thus, the expression profiles of circRNAs in serum were also obtained to seek a potential biomarker for the diagnosis of RPL. Previous studies have suggested some circRNAs in blood corpuscles or whole blood that might be potential biomarkers for pre-eclampsia based on the expression analysis of the circRNAs in more than 60 samples [31,37]. CircRNA expressions in sera from six samples were detected in the present study. Thus, the limited sample size and differences in sample composition might have effects on the expression profiles of circRNAs and might be the reason for the lack of detection of differentially expressed circRNAs in the serum between the NP and RPL groups. Previous research has identified that m6A-modified hsa_circ_0000816 promotes GLUT1 mRNA stability in oral squamous cell carcinoma [38]. Whether it regulates glycolysis in pregnancy and is a biomarker for RPL needs further clarification. Although the expression trends of some circRNAs in serum were similar to those in the decidua, multicenter, many more participants and sample multicomponent analysis are needed to investigate reliable biomarkers of RPL in future study.

## 5. Conclusions

**Figure 6:**
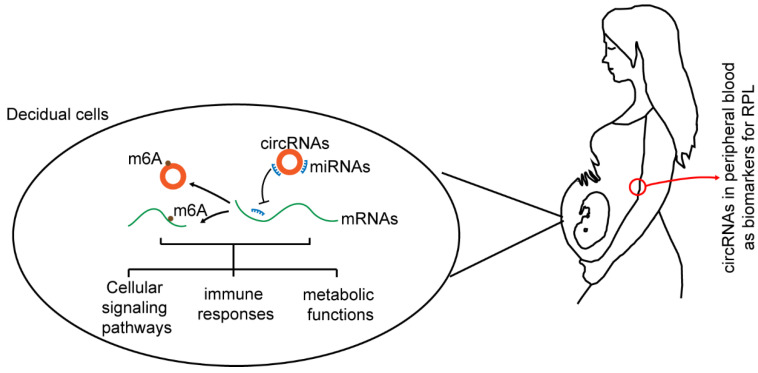
Schematic diagram showing the association between circRNAs and RPL. The circRNAs might participate in management of signaling, immune and metabolism related pathways by regulating ceRNA network and m6A modification to maintain pregnancy. Seeking several circRNAs in peripherial blood as potential biomarkers for RPL might provide assistance in exploring the pathogenesis and diagnosis of RPL.

We displayed the expression profiles of circRNAs and their potential functions in signaling, immune-related and metabolism-related pathways. The possible pathogenesis of RPL was explored in the aspect of functional analysis of a ceRNA network and m6A modification. This revealed the potential relationship between decidual circRNAs in RPL and abnormal m6A RNA modifications (Figure 6). Our study might provide assistance in further research into m6A modification and in exploring the pathogenesis and diagnosis of RPL.

## Figures and Tables

**Table 1:** Clinical characteristics of enrolled subjects.

Subjects ^b^	NP (n = 9)	RPL (n = 9)	*p*-Value
Age mean (years) ^a^	29.89 ± 2.43	30.33 ± 1.63	0.88
Age range (years)	22–40	23–36	-
Previous pregnancy loss ^a^	0	2.778 ± 0.28	<0.0001
Previous normal births ^a^	1.444 ± 0.24	0	<0.0001
Pregnancy week (sample collected) ^a^	6.21 ± 0.24	6.43 ± 0.14	0.44

^a^ Values are expressed as the median ± standard error of the mean. RPL was defined as spontaneous abortion in patients who also had a history of two or more consecutive pregnancy loss without known causes. ^b^ None of the subjects had any history of treatment. Groups: NP, normal early pregnancy; RPL, recurrent pregnancy loss.

**Table 2:** Primers used for RT-PCR.

Primer Name	Forward	Reverse
hsa_circ_0002535	CTCCTTGGCACTGTGCTTCC	GAGCCCAGCAGTTGCCC
hsa_circ_0065307	CAAACGACATCAGGCAAAGTGT	GTAGGTCAAACCTCCGCCAT
hsa_circ_0093528	ACAGATGGCGTAATGGGGTG	AACTGCTACATGTCTGTTGGA
hsa_circ_0116552	CTCGAAGGATGCGCAGAGAT	GCAGATCTCAACACCATTAAGTACC
hsa_circ_0125759	GGAAGTGAAGAGGCTGACATGA	GGAAGTGAAGAGGCTGACATGA
hsa_circ_0005741	GGTCCATTGCTATCAGCCCA	CCTTCACTGGGACACTGGTC
hsa_circ_0008129	AGCTACTTGTAGAGGCTTATTGTGT	AGCTACTTGTAGAGGCTTATTGTGT
hsa_circ_0009049	CCTTCTTCTCTGGCCATGC	ACTCAAGAAGTGAGGACGCA
hsa_circ_0074945	TCTACGACCCCAACAAGCAA	AGCTCAAGGATTCGTGAAGACC
hsa_circ_0115550	GAAACACAGGACATCGCTGC	AGACACTGAAGATCAGGCCAAC

## Data Availability

The datasets used and/or analyzed during the current study are available from the corresponding author on reasonable request.

## Supplementary Materials

The following supporting information can be downloaded at: https://www.mdpi.com/article/10.3390/cimb45110551/s1, Figure S1: categories and length of differentially expressed circRNAs. (A) categories of differentially expressed circRNAs in decidua between NP groyup and RPL group. (B) length of differentially expressed circRNAs in decidua between NP group and RPL group; Table S1: KEGG pathway of parental genes of circRNAs.
