# Supplementary material for: Identification of m6A Modification Regulated by Dysregulated circRNAs in Decidua of Recurrent Pregnancy Loss
Source: Curr Issues Mol Biol. 2023 Oct 31;45(11):8767–79. doi: 10.3390/cimb45110551 (PMC10670759; doi:10.3390/cimb45110551)
Supplement: Supplementary file 1 [file cimb-45-00551-s001.zip › cimb-2660463-supplementary.pdf]

Figure S1. Categories and length of differentially expressed circRNAs. (**A**) categories of differentially expressed circRNAs in decidua between NP groyup and RPL group. (**B**) length of differentially expressed circRNAs in decidua between NP group and RPL group.

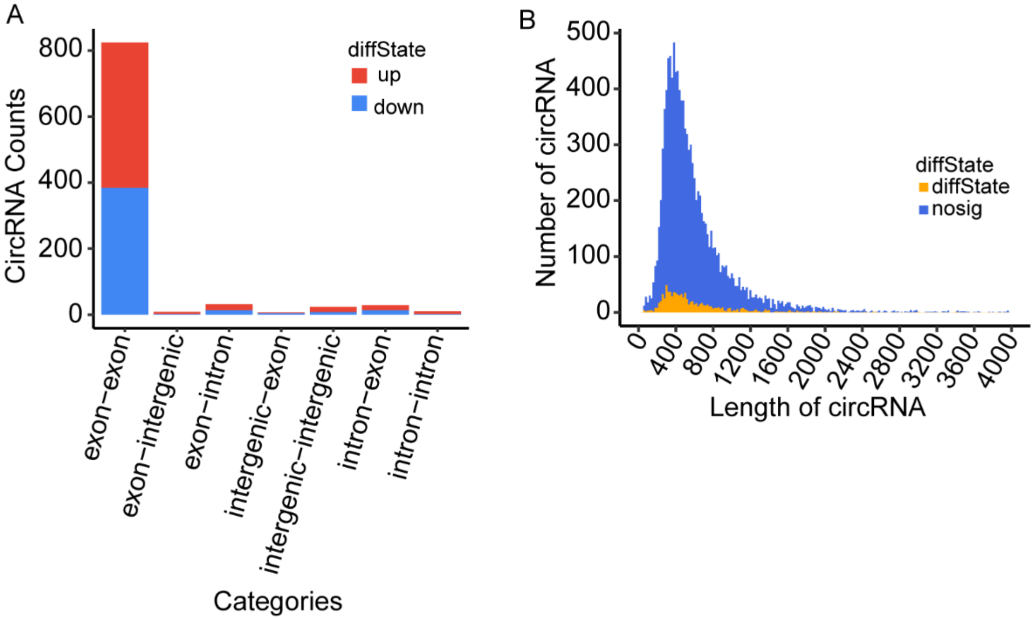

Table S1. KEGG pathway of parental genes of circRNAs.

databaseID	Description	geneRatio	bgRatio	pvalue	padj	enrichScore	overlapGeneList	overlapGeneCount
hsa00310	Lysine degradation	9/109	63/2426	0.0016	0.0761	3.1795	ASH1L/PLOD2/PRDM2/SMYD2/DOT1L/SETD2/NSD2/NSD1/EHMT1	9
hsa01521	EGFR tyrosine kinase inhibitor resistance	10/109	79/2426	0.0023	0.0761	2.8173	AKT3/NF1/RPS6KB1/AKT2/PIK3CA/PDGFRA/BRAF/PLCG2/STAT3/MAP2K2	10
hsa00532	Glycosaminoglycan biosynthesis - chondroitin sulfate /	4/109	20/2426	0.0106	0.1738	4.4513	CSGALNACT1/CHSY1/XYLT1/CHST12	4

	dermatan sulfate							
hsa00670	One carbon pool by folate	4/109	20/2426	0.0106	0.1738	4.4513	GART/MTHFD1L/MTHFD1/MTHFD2L	4
hsa00562	Inositol phosphate metabolism	8/109	73/2426	0.0150	0.1960	2.4391	PIK3C3/PIK3CA/PIP5K1A/INPP5B/INPP5A/PI4K2A/PLCG2/PIKFYVE	8
hsa01522	Endocrine resistance	9/109	98/2426	0.0294	0.3194	2.0439	AKT3/RPS6KB1/AKT2/PIK3CA/BRAF/PTK2/NCOR1/MAP2K2/MAPK10	9
hsa01523	Antifolate resistance	4/109	31/2426	0.0476	0.3194	2.8718	CHUK/ABCC1/GART/ABCC5	4
hsa03015	mRNA surveillance pathway	8/109	98/2426	0.0700	0.5691	1.8168	PPP2R1B/PPP2R5C/PAPOLG/RNGTT/UPF2/DDX19A/PPP2R3A/NCBP1	8
